# Phthalates as the silent saboteurs of male fertility via changes in semen quality: a systematic review

**DOI:** 10.1186/s12958-026-01541-0

**Published:** 2026-03-09

**Authors:** Swati Dhar, Akash Tomar, Anupama N, Poulomi Chatterjee, Pratik Kumar Chatterjee

**Affiliations:** 1 Department of Reproductive Medicine and Surgery, Kasturba Medical College, Manipal Academy of Higher Education, Manipal, Karnataka India; 2 Department of Physiology, Kasturba Medical College, Manipal Academy of Higher Education, Manipal, Karnataka India; 3 Department of Physiology, Kasturba Medical College Mangalore, Manipal Academy of Higher Education, Manipal, Karnataka India; 4 Department of Community Medicine, Yenepoya Medical College, Yenepoya (Deemed to be University), Deralakatte, Mangalore, Karnataka India

**Keywords:** Phthalate exposure, Male infertility, Sperm quality, Endocrine disruptors, Environmental health, Reproductive toxicity, Sustainable fertility

## Abstract

**Background:**

Phthalates, which are commonly used as plasticizers, are pervasive in modern environments. The potential effects of endocrine-disrupting chemicals (EDCs) on human reproductive health have raised concerns. Among the various health risks, a significant focus has been on male fertility, particularly the relationship between phthalate exposure and a reduced sperm count. Di-(2-ethylhexyl) phthalate (DEHP) and di-n-butyl phthalate (DBP) are two of the most studied phthalates because of their wide use in products such as plastics, cosmetics and medical devices. Growing evidence suggests that they may impair sperm production, viability, and quality, contributing to male infertility. This review synthesizes epidemiological and experimental evidence on the reproductive toxicity governed by phthalates in semen parameters.

**Methods:**

This systematic review was conducted following the SWiM (synthesis without meta-analysis) guidelines. A comprehensive search across three major databases was conducted to capture literature on phthalate exposure and semen parameters, including sperm concentration, morphology and motility, published between 2014 and 14/10/2024. Two independent researchers screened and selected studies. The risk of bias in the included studies was assessed via ROBINS-E tool. A meta-analysis was not performed due to variability in the study designs.

**Results:**

38 studies met the eligibility criteria and were included in this review. Human phthalate exposure has shown that semen parameters are altered, such as a decrease in the sperm concentration. Research suggests that high exposure to phthalates such as MEHP, DEHP and DBP is associated with reduced sperm concentration, motility, and overall semen quality. Phthalates remain a potential contributing factor in male fertility issues, but the variability in findings across populations and exposure levels in these populations indicate that more research is needed to understand this relationship.

**Conclusion:**

Phthalates, particularly MEHP, DEHP and DBP, negatively affect sperm concentration and affect sperm morphology, likely through endocrine disruption and impairment of testicular function via oxidative stress. The certainty of the evidence is graded as low to moderate, primarily due to the observational nature of the included studies. However, given the potential risks to reproductive health, the evidence supports a precautionary approach advocating stricter regulation of phthalate exposure in everyday products.

**Supplementary Information:**

The online version contains supplementary material available at 10.1186/s12958-026-01541-0.

## Introduction

Phthalates are a group of industrial chemicals widely used in the manufacturing of plastics, particularly polyvinyl chloride (PVC). These chemicals make plastics more flexible, durable, and difficult to break [1], and they are also commonly found in everyday products, including food packaging and medical devices [2]. Despite their utility, phthalates have drawn significant attention because of their potential adverse effects on human health, particularly in terms of male fertility. In the last few decades, evidence has accumulated linking exposure to phthalates with disruptions in male reproductive health, particularly affecting semen quality, sperm concentration, motility, and morphology [3–5].

### Phthalates: Overview and sources of exposure

Phthalates are considered endocrine-disrupting chemicals (EDCs) that interfere with the hormonal regulation system of the human body [6]. The two primary forms of phthalates most commonly studied for their impact on male fertility are di(2-ethylhexyl) phthalate (DEHP) and di-n-butyl phthalate (DBP). These compounds are prevalent in a variety of consumer products, such as personal care products (lotions, shampoos), food containers, medical devices, and even indoor environments, through the degradation of building materials [7–10].

Human exposure to phthalates is nearly unavoidable due to their omnipresence in modern life. Phthalates can leach into food, water, and air, leading to ingestion, inhalation, or dermal absorption [11–13]. Once inside the human body, phthalates are metabolized and excreted, but studies have reported measurable concentrations of phthalate metabolites in human urine, blood, and seminal fluid, even in individuals without known occupational exposure [14, 15]. These findings have raised concerns about their long-term effects on health, particularly in vulnerable populations such as pregnant women and men of reproductive age.

### The role of phthalates as endocrine disruptors

Phthalates act as endocrine disruptors by mimicking or interfering with the function of hormones in the body [16–18]. In men, phthalates can interfere with the production of testosterone, a key hormone responsible for regulating sperm production and overall male reproductive health [4, 19, 20]. Studies suggest that phthalates may inhibit the activity of enzymes involved in testosterone synthesis, leading to reduced testosterone levels and impaired spermatogenesis [21, 22]. Research on phthalates also indicates that they may influence other hormone-related processes. For example, phthalates have been linked to changes in luteinizing hormone (LH) and follicle-stimulating hormone (FSH) levels, both of which are critical for reproductive function [23–25]. Disruptions in these hormones can lead to a variety of reproductive issues, including decreased sperm count, reduced sperm motility, and abnormal sperm morphology.

The precise biological mechanisms by which phthalates affect semen quality are not fully understood, but several hypotheses have been proposed. One of the most widely accepted mechanisms involves the generation of oxidative stress. Phthalates are believed to induce oxidative stress in the testis, leading to cellular damage [26–28]. The delicate balance of ROS and antioxidants is essential for normal sperm production and function. Excessive ROS can damage sperm DNA, impair mitochondrial function, and alter cell membrane integrity, all of which can lead to impaired semen quality [29, 30].

Another potential mechanism is the disruption of testosterone synthesis. Phthalates are known to inhibit the activity of enzymes such as 17β-hydroxysteroid dehydrogenase, which is crucial for converting precursors into testosterone [31]. Reduced testosterone levels can impair spermatogenesis and contribute to various sperm abnormalities. In addition to these mechanisms, phthalates may also interfere with gene expression related to reproductive function. Epigenetic modifications, such as DNA methylation and histone acetylation, have been observed in animal studies, suggesting that phthalates may induce long-lasting changes in the genetic regulation of sperm production and quality [32].

### Phthalates and semen quality: current research

#### Sperm concentration

The sperm concentration refers to the number of sperm cells present in a given volume of semen. A decrease in the sperm concentration is one of the most concerning trends associated with male infertility. Numerous studies have suggested a link between phthalate exposure and a reduced sperm concentration. For example, a meta-analysis published by Radke et al. [4] reported moderate evidence supporting the association between increased DEHP exposure and decreased sperm concentration, implying that higher levels of phthalates in the body could contribute to lower sperm counts.

A global decline in sperm concentration has been documented, with studies reporting a 50–60% reduction in sperm counts among men from the 20th to 21st centuries [33]. While lifestyle factors such as diet and stress undoubtedly play a role in this decline, the widespread presence of phthalates in modern environments suggests that these chemicals may contribute to the problem. Some studies, however, report only weak associations between phthalate exposure and sperm concentration, indicating that more research is needed to establish a definitive causal link [4, 34, 35]. However, experimentally induced phthalate exposure in animal models has always shown a negative effect on the sperm concentration [36].

### Sperm motility

Sperm motility is a functional measurement of the sperm themselves. It also refers to the ability of sperm to move efficiently through the female reproductive tract to fertilize an egg. Reduced motility is another factor strongly associated with male infertility [37]. Phthalates, particularly DEHP, have been shown to negatively affect sperm motility [38]. The mechanism through which this occurs is thought to involve oxidative stress induced by phthalates. By disrupting the normal balance of reactive oxygen species (ROS) and antioxidants [39], phthalates can cause damage to the mitochondria of sperm cells [40], which is critical for providing the energy required for sperm movement.

#### Sperm morphology

Sperm morphology refers to the size and shape of sperm cells. Among all individual semen parameters, abnormal morphology is associated with the highest risk of infertility (concentration, motility, and morphology) [41]. Sperm morphology is critical for fertility, as irregularly shaped sperm may struggle to swim through the cervical mucus and penetrate the egg’s outer layers. Like sperm concentration and motility [26], oxidative stress is believed to play a role in the morphological abnormalities caused by phthalates [42]. Phthalates, especially DBP, are also associated with an increase in the percentage of sperm with abnormal morphology [43]. Radke et al. [4] noted that greater exposure to phthalates corresponded with increased rates of sperm exhibiting abnormal shapes and structures, which could further reduce the likelihood of successful fertilization, even with the use of ART. Even in the setting of IVF/ICSI, which bypasses oligoasthenozoospermia, abnormal morphology still remains detrimental to fertilization potential, thereby affecting reproductive outcomes.

While phthalate exposure has been linked to reduced semen quality in many studies, it is important to recognize that the impact of these chemicals can vary across populations [44]. Factors such as age, lifestyle, genetic predispositions, and overall health status can influence the extent to which phthalates affect male fertility [45, 46]. Occupational exposure is also a significant factor; individuals working in industries that involve the handling of plastics, paints, and other materials containing phthalates are likely to experience higher levels of exposure and, consequently, more pronounced effects on their reproductive health [47]. Geographical differences also play a role. Studies have indicated that men from industrialized nations, particularly in Western countries, are more likely to experience the reproductive effects of phthalates due to higher environmental concentrations of these chemicals [48]. In contrast, men from less industrialized regions may face lower levels of exposure but could still be at risk depending on local environmental and occupational factors.

Given the growing body of evidence linking phthalates to male reproductive issues, several countries have implemented regulatory measures aimed at reducing human exposure to these chemicals. For example, the European Union has restricted the use of certain phthalates, including DEHP and DBP, in consumer products such as toys and cosmetics [49]. Similarly, the United States has imposed limits on the concentration of phthalates in products intended for children [50]. These regulatory actions are based on the principle of reducing exposure to endocrine disruptors, particularly among vulnerable populations such as pregnant women and children. Despite these efforts, phthalates remain prevalent in the environment, and mitigating their impact on public health requires more comprehensive strategies. This includes increasing public awareness of the potential risks associated with phthalates, encouraging the development of safer alternatives in manufacturing, and enhancing biomonitoring efforts to better understand the scope of human exposure. The link between phthalate exposure and male fertility, particularly in terms of semen quality, is a critical public health issue. Given the widespread use of phthalates in modern life, reducing exposure to these chemicals is crucial for safeguarding male reproductive health. Although regulatory efforts have been made in many countries, continued research, public education, and policy reforms are necessary to address the full scope of the problem and minimize the impact of phthalates on fertility.

This systematic review explores how phthalates influence male fertility, focusing specifically on their impact on semen quality. We will examine current research on the subject, scrutinize various types of phthalates that are commonly associated with these effects, and explore the biological mechanisms behind this disruption.

## Methods

This systematic review was conducted following the criteria of synthesis without meta-analysis in systematic reviews SWiM [51]. A PRISMA 2020 Checklist [52] is also attached as supplementary file 1 (S1). The systematic review is registered in the open science framework (https://osf.io/a6n4b) [53].

### Expert consultation

Prior to developing the search strategy, an expert consultation was performed to refine the preliminary research questions of the review through the collation of feedback received from stakeholders and public health experts in the context of exposure to a toxic environment of phthalates, male fertility and semen parameters.

### Eligibility criteria

The review included randomized controlled trials (RCTs), nonrandomized trials/quasi-experimental studies, cluster randomized trials, repeated measures studies, interrupted time series studies, case‒control studies, cross-sectional studies, interventional studies and controlled before‒after studies. Studies reporting exposure to phthalates in men and any type of change in semen parameters, most notably sperm count, were selected. Only studies published in English were considered. The publication time of the review was limited to the period from 2014 to 14th October 2024. Studies published as editorials, letters, opinions, brief communications, or short reports were excluded. The search strategy was developed on the basis of the following population, intervention, comparison, and outcome (PICO) model.

### Population

Adult men from the general population, subfertile/infertile clinical cohorts, and occupationally exposed groups included in human biomonitoring or environmental exposure studies.

### Exposure

Environmental or biomonitoring-based exposure to phthalates, assessed through urinary metabolites, blood levels, or other validated biomarkers indicating internal dose.

### Comparison

No comparison was performed in this review.

### Outcome

Alterations in sperm quality parameters, including sperm concentration, motility, morphology, and other semen characteristics related to male reproductive function.

### Data sources and searches

We searched three major electronic databases (PubMed, Scopus and Web of Science), as most of the articles pertaining to our review topic were found in these three databases. The search through the electronic databases was conducted on 14th October 2024. An updated and comprehensive search strategy was developed using key terms and synonyms such as title-abs-key (phthalate and sperm and count) AND (limit-to (doctype, “ar”)) AND (limit-to (exactkeyword, “male”) OR limit-to (exactkeyword, “spermatozoon count”) OR limit-to (exactkeyword, “human”) OR limit-to (exactkeyword, “sperm count”) OR exclude (exactkeyword, “animals”) OR exclude (exactkeyword, “animal experiment”) OR exclude (exactkeyword, “female”) OR exclude (exactkeyword, “rat”) OR exclude (exactkeyword, “animal tissue”) OR exclude (exactkeyword, “rats”) OR exclude (exactkeyword, “animal model”) OR exclude (exactkeyword, “animalia”) OR exclude (exactkeyword, “animal”) OR exclude (exactkeyword, “animal cell”) AND (limit-to (language, “english”) for Scopus. An updated and comprehensive search strategy was developed using key terms and synonyms such as ((“phthalates“[All Fields] OR “phthalic acid“[Supplementary Concept] OR “phthalic acid“[All Fields] OR “phthalate“[All Fields]) AND “sperm s“[All Fields] OR “spermatozoa“[mesh Terms] OR “spermatozoa“[All Fields] OR “sperm“[All Fields] OR “sperms“[All Fields]) AND (“count“[All Fields] OR “counted“[All Fields] OR “counting“[All Fields] OR “countings“[All Fields] OR “counts“[All Fields])) AND ((y_10[Filter]) AND (humans[Filter]) AND (male[Filter]) AND (english[Filter])) was used for PubMed. An updated and comprehensive search strategy was developed using key terms and synonyms such as was (phthalate OR phthalates OR plasticizer* OR endocrine disrupt*) (All Fields) AND (male OR men OR testis OR sperm OR semen OR reproduction) (All Fields) used for Web of science. Several relevant articles have been reviewed and cross-checked to assess the comprehensiveness of the search strategy. According to our review question, study selection and data extraction, the articles retrieved from the initial search were exported in Rif format for Scopus, PubMed and Web of science and then moved to Rayyan for sorting and reviewing. Then, duplicates were removed, and the studies were screened by title and abstract. The studies were subsequently screened by full text and selected for data extraction following the inclusion and exclusion criteria. Four reviewers who were divided into two groups were involved in the screening process. Two reviewers independently screened the included studies by title and abstract and, accordingly, full text. The lead reviewer was involved in resolving any kind of dispute. The systematic review was conducted following SWiM guidelines. Two reviewers independently extracted data via a data extraction form containing basic data that included the following criteria: author, publication year, publication type (e.g., original research), study design and outcome assessment. Any disagreement between the reviewers was resolved through discussion with the lead reviewer, and an opinion was taken from the review team if necessary.

### Quality assessment

Two reviewers independently assessed the risk of bias. Any uncertainties and discrepancies were resolved by discussion, further review of the respective study, and consultation with a third reviewer wherever deemed necessary. The risk of bias assessment was adopted from the criteria outlined in the Cochrane Handbook for Systematic Reviews of Interventions and Effective Public Health Practice Project (EPHPP) checklist, which is the ROBINS (Risk Of Bias In Nonrandomized Studies) [54]. In this review, “blinding” was not considered a quality assessment criterion, as the blinding of participants or intervention implementers is rare in community-based interventions. The items “random sequence generation” and “allocation concealment” were not considered when assessing the cohort studies included in this review.

### Data analysis and synthesis

Studies reporting the effects of phthalate exposure on semen quality and other sperm parameters were included. As the main outcome, data on the sperm count, morphology, motility and other characteristics were extracted. In addition, data on the type of phthalate causing these effects were also extracted. The extracted data were carefully read, and information regarding the disruption of semen quality due to phthalate exposure was obtained. Meta-analysis was not possible because of significant heterogeneity in the comparison groups, outcomes of interest, outcome measurements, type of study design and statistical analysis. Instead, a descriptive analysis of the study findings was performed.

## Results

### Literature review

**Fig. 1:**
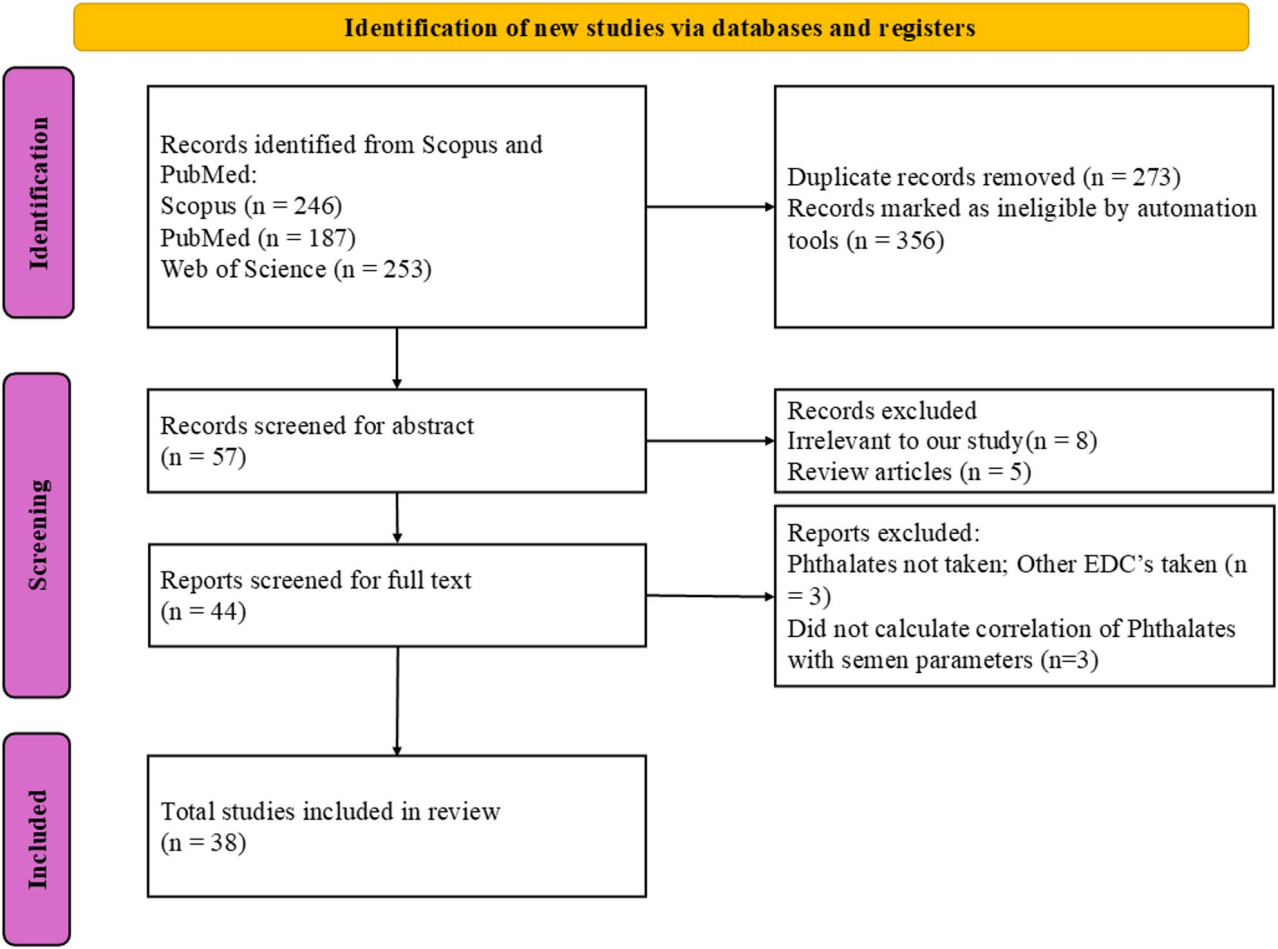
PRISMA flow diagram for data identification, screening and inclusion [52]

Figure 1 shows the flow chart detailing the literature search and selection process. Initially, a total of 686 records were identified through database scanning. After removing duplicates and utilizing automation tools, 54 records were retained for further consideration. Subsequently, epidemiological studies lacking full-text availability, as well as specific types of publications (such as reviews, commentary articles, meta-analyses, and animal or molecular experimental research), were excluded. This refinement left 44 records for full-text evaluation.

Among these, three studies were excluded because they focused on other endocrine-disrupting chemicals (EDCs), such as bisphenol and organophosphate esters, rather than phthalates [55–57]. Additionally, three studies were excluded because they examined the relationships between phthalates and other reproductive hormones (e.g., LH, FSH, and testosterone) but did not address semen parameters [20, 58, 59]. Ultimately, 38 studies were included as the primary focus of the current systematic review [35, 47, 60–94].

### Characteristics of the included studies

Table 1 Inventory research information and outcome measures for 38 studies. The final dataset involved 25 cross-sectional studies, 7 prospective, 5 case‒control and 1 retrospective cohort. The 38 studies involved different sample types, including urine, serum and semen samples from males. Four studies examined phthalate exposure and its correlation with semen quality. Researchers have performed either high-pressure liquid chromatography‒tandem mass spectrometry (HPLC‒MS/MS) or liquid chromatography‒tandem mass spectrometry (LC‒MS/MS) to determine the concentration of phthalate metabolites in biological samples in nearly all included studies. Owing to the difficult combination of measurements and significant variations in the statistical description of outcome measures, it was not possible to perform a meta-analysis in this systematic review.

**Table 1:** Characteristics of the studies included in the Systematic Review

Author and Year	Study design	Sample size	Country	Semen Parameters evaluated	Measurement of phthalates in	Conclusions
Gao et al. 2024 [61]	Prospective	155	China	Volume Concentration Total count Total motillity Progressive motility	Urine	Higher urinary MBP was linked to a 23.7% decrease in seminal volume. MCPP consistently showed harmful effects on semen quality and was prioritized for deeper analysis.
Liu et al. 2019 [62]	Cross-sectional	1034	China	Concentration Total count Total motillity Progressive motility Normal morphology	Urine	Urinary MBP was inversely associated with sperm concentration, while urinary MEP was linked to reduced normal sperm morphology.
Caporossi et al. 2023 [63]	Case‒control	366	Italy	Volume Concentration Total count Total motillity	Urine	Higher exposure to multiple phthalates was observed in infertile men compared to their fertile counterparts.
Rana et al. 2020 [64]	Case‒control	227	India	total count viscosity motility morphology vitality	Urine, Serum	Infertile males had significantly higher phthalate concentrations, although no direct associations with semen quality parameters were observed.
Pan et al. 2015 [65]	Cross-sectional	1066	China	Volume Concentration Count Morphology motility Acrosin activity	Urine	MBP, MiBP, and MEHP were linked to altered acrosin activity, while MBP and MiBP were also associated with abnormal sperm morphology.
Specht et al. 2014 [66]	Cross-sectional	589	Denmark	Volume Total count Concentration morphology motility	Serum	Proxy-MEHP was associated with reduced semen volume and total sperm count, but not with sperm concentration. Higher levels of 5OH-MEHP were linked to declines in semen volume and total sperm count. No associations were observed between serum phthalate metabolites and sperm concentration, morphology, or motility.
Pan et al. 2016 [67]	Cross-sectional	562	China	Volume Concentration Count Progressive motility Morphology	Urine	Inverse associations were observed between phthalate levels and sperm concentration, total sperm count, and morphology.
Broe et al. 2018 [68]	Case‒control	49,578	Denmark	Semen quality	Phthalate Exposure	Phthalate exposure was associated with negative effects on overall semen quality.
Nassan et al. 2016 [69]	Crossover-crossback prospective	73	USA	Volume Sperm concentration Total sperm count Total sperm motility % Progressive sperm motility % Motile sperm count % Normal sperm morphology Morphologically normal sperm count	Phthalate Exposure	Men newly exposed to high-DBP mesalamine for four months showed cumulative declines in semen parameters, especially sperm motility.
Hond et al. 2015 [70]	Case‒control	163	Belgium	Sperm concentration Motility Normal motility Morphology TMC	Urine	No direct association was found between phthalates and semen quality, but phthalates were linked to altered serum LH and inhibin levels, suggesting disruption of Leydig and Sertoli cell function.
Yang et al. 2017 [71]	Cross-sectional	473	China	Sperm Concentration Sperm count Sperm Motility	Urine	Apoptosis-related gene polymorphisms may influence phthalate effects on male reproductive health, offering insights into the underlying causes of male infertility.
Ramsay et al. 2023 [72]	Retrospective cohort study	21,563	USA	Concentration (M/mL) Ejaculate volume (mL) Total count (M) Total motility (%) Total motile count (M) Total progressive motile count (M)	Phthalate Exposure	Phthalate exposure was associated with increased odds of azoospermia (OR = 1.44) and reduced semen volume (β = −0.09 mL) and total motility (β = −1.21% points).
Jurewicz et al. 2016 [73]	Cross-sectional	194	Poland	Concentration Morphology Motility	Urine	Exposure to endocrine-disrupting chemicals (EDCs) was associated with a decreased Y/X sperm chromosome ratio.
Al-Saleh et al. 2019 [95]	Prospective	599	Saudi Arabia	%DNA damage	Urine	An increase in urinary MEP levels was linked to a 13.7% rise in sperm total motility, although the association was only marginally significant.
Albert et al. 2018 [75]	Cross-sectional	153	Canada	Sperm Concentration Sperm motility %DNA comet Tail	Urine	No correlation was found between phthalate exposure and sperm parameters.
Chen et al. 2017 [76]	Prospective	796	China	Volume Concentration Count Motility Morphology	Urine	Urinary phthalate metabolites were associated with changes in semen and hormone outcomes. A comprehensive reduction in phthalate exposure was linked to improvements in these reproductive parameters.
Yuan et al. 2021 [77]	Cross-sectional	1319	China	semen volume Sperm concentration, count, motility parameters motion parameters	Semen	Inverse associations were found between semen MnBP levels and multiple semen quality parameters, as well as certain seminal plasma steroids. Androgen disruption (ADD) potentially mediated approximately 10% of the observed link between MnBP and reduced sperm motility.
Thurston et al. 2016 [78]	Cross-sectional	420	USA	Concentration motility Count morphology Total motile count	Urine	No associations were found between adult phthalate exposure and standard semen quality parameters in fertile U.S. men. The exception was MBzP, which showed a negative association with sperm motility.
Yang et al. 2023 [79]	Cross-sectional	909	China	Volume Concentration Total count Total motillity Progressive motility Sperm Telomere length Sperm mtDNA copy no.	Urine	Elevated PAE levels during spermatogenesis, particularly MEHP, were linked to reduced sperm concentration and motility. Mediation analysis indicated that sperm mtDNA copy number may partly mediate the effects of PAE exposure on semen quality.
Wang et al. 2015 [80]	Cross-sectional	1040	China	Volume Concentration Total count Total motillity Progressive motility Motion Morphology	Urine	DBP and DEHP exposure may contribute to a decline in semen quality.
Axelsson et al. 2015 [81]	Cross-sectional	314	Sweden	Volume Concentration Total count Progressive motility Morphology High DNA stainability DNA fragmentation index	Urine, Serum	Higher DEHP metabolite levels were linked to reduced proportions of progressively motile and mature spermatozoa.
Wang et al. 2020 [60]	Cross-sectional	88	China	Volume Concentration Total count Total motillity Progressive motility	Serum	Positive associations were observed between serum mPAE concentrations and semen parameters. Mediation analysis confirmed that FSH and LH partially mediated the relationship between certain mPAEs and semen quality, highlighting endocrine-disruptive mechanisms.
Liu et al. 2017 [82]	Cross-sectional	289	China	Volume Concentration Motility Velocity (many types)	Urine	MEHHP levels were significantly higher in infertile men compared to fertile men.
Tian et al. 2018 [83]	Cross-sectional	86	China	Volume Concentration Total count motillity Morphology	Urine	Phthalate exposure was linked to reduced sperm motility in clinically subfertile males.
Pant et al. 2014 [84]	Cross-sectional	60	India	Concentration motillity Morphology %DNA tail	Semen	Phthalates DEHP, DEP, and DBP may independently reduce semen quality and cause sperm DNA damage.
Mínguez-Alarcón et al. 2018 [85]	Prospective	1482	USA	Sperm concentration total count total motility	Urine	Including DEHP and non-DEHP metabolites attenuated the downwards trends in sperm concentration and morphology over time by 19%.
Wang et al. 2016 [86]	Cross-sectional	687	China	Volume Concentration Total count Total motillity Progressive motility Morphology Motion/velocity	Semen	Semen phthalate metabolites MBP, MEHP, MEHHP, and MEOHP were associated with decreased semen volume. MBzP, MEHP, and %MEHP showed inverse associations with sperm velocity parameters VCL and VSL. MBzP was linked to a higher percentage of abnormal sperm heads and tails, indicating semen quality impairment from environmental BBzP, DBP, and DEHP exposure.
Huang et al. 2019 [87]	Cross-sectional	98	China	Volume Concentration Total count Total motillity Progressive motility Morphology	Urine	MEHP positively associated with Sperm count
Wang et al. 2019 [88]	Cross-sectional	660	China	Volume Concentration Total count Total motillity Progressive motility Morphology	Urine	Mediation analysis revealed that oleic acid and L-acetylcarnitine significantly mediated the positive association between urinary DEHP metabolites and abnormal sperm head morphology.
Wang et al. 2018 [89]	Cross-sectional	509	China	Volume Concentration Total count Total motillity Progressive motility Morphology	Urine	Higher urinary MEP and %MEHP were linked to reduced serum thyroid hormones (FT4, FSH), which were in turn associated with impaired semen quality. Mediation analysis showed that FT4 significantly mediated the inverse relationship between urinary MEP and normal sperm morphology.
Bloom et al. 2015 [35]	Prospective cohort	501	USA	Volume Concentration Total count Total motillity Progressive motility Morphology Motion/velocity	Urine	Phthalate diesters may impair semen quality even at low exposure levels commonly found in the general population.
Huang et al. 2014 [47]	Case control	62	Taiwan	Volume Concentration Motility Morphology ROS generation Apoptosis	Urine	Urinary MEHP concentration showed no significant association with sperm apoptosis.
Deng et al. 2022 [90]	Cross-sectional	695	China	Concentration Count Motility Progressive motility	Urine	DEHP exposure showed a nonmonotonic association with increased progressive and total sperm motility. Overall, DEHP was linked to altered semen quality, highlighting complex dose–response dynamics.
Al-Saleh et al. 2019 [74]	Cross-sectional	599	Saudi Arabia	Cocentration Volume motility morphology	Urine	Higher exposure to DEHP metabolites (MECPP, MEHHP, MEOHP, ΣDEHP) was associated with a reduced risk of very low sperm concentration (≤ 15 million/mL). However, men with a higher proportion of MEHP relative to other DEHP metabolites may still be at risk for low sperm concentration.
Mínguez-Alarcón et al. 2022 [92]	Prospective	223	Russia	Volume Concentration Total count Total motillity Progressive motility	Urine	Higher urinary ΣDiNP concentrations in late puberty were linked to poorer semen quality at sexual maturity. Elevated MiBP levels during early puberty also showed an association with reduced semen quality. No consistent associations were observed for ΣDiNP or MiBP at other pubertal stages, or for ΣDEHP, ΣDiDP, and ΣAAP at any point during puberty.
Han et al. 2014 [93]	Cross-sectional	232	China	Concentration Count Volume motility morphology	Urine	A weak inverse association was observed between urinary MBP concentration and sperm concentration in the general male population of the geography.
Chang et al. 2015 [20]	Cross-sectional	273	Taiwan	Volume Concentration motility morphology liquefaction	Urine, Semen	Urinary and seminal metabolites of DEP, BBzP, and DEHP were jointly associated with reduced INSL3 and testosterone levels. These exposures were also linked to impaired sperm production and decreased semen quality in adult males.

To address the heterogeneity in exposure assessment methods, we stratified the included studies by biospecimen type and the timing of measurement relative to the spermatogenic cycle (Table 2). Many studies (*n* = 22) relied on single spot urine samples collected concurrently with semen analysis. While urinary metabolites are the standard biomarker for phthalate exposure, single-point measurements primarily reflect recent exposure (hours to days) and may not fully capture the exposure burden during the critical 70-to-90-day window of spermatogenesis as shown in Table 2. However, four studies utilized longitudinal or repeated-measures designs providing stronger evidence for causal inference by linking specific exposure windows such as puberty or defined pharmaceutical use to subsequent changes in semen quality as shown in Table 2. A smaller subset of studies quantified phthalates in serum or directly in seminal plasma, offering insight into the internal dose reaching the reproductive tract. Axelsson et al. [81] and Rana et al. [64] utilized multiple matrices (Urine/Serum); they are grouped here by their primary contribution.

**Table 2:** Biological matrices and study designs used to assess phthalate exposure in relation to male spermatogenesis

Exposure Matrix	Study Design	Study Reference	Timing of Assessment Relative to Spermatogenesis
Urine (Metabolites)	Longitudinal / Repeated Measures	Mínguez-Alarcón et al. (2022) [92]	Developmental Windows: Annual sampling pooled into four windows (pre-puberty to sexual maturity); assessing long-term impact on spermatogenesis.
		Chen et al. (2017) [76]	Repeated Measures: Samples collected before and after environmental relocation to assess changes in exposure and semen quality over time.
		Mínguez-Alarcón et al. (2018) [85]	Repeated Measures: Mixed models accounting for repeated semen and urine samples over the study period (2000–2017).
		Nassan et al. (2016) [69]	Crossover-Crossback: Samples collected during a defined 4-month exposure to DBP-containing mesalamine (covering one spermatogenic cycle) vs. non-exposure periods.
Urine (Metabolites)	Cross-Sectional / Single Point	Gao et al. (2024); Liu et al. (2019); Caporossi et al. (2023); Pan et al. (2015); Pan et al. (2016); Den Hond et al. (2015); Yang et al. (2017); Jurewicz et al. (2016); Al-Saleh et al. (2019); Albert et al. (2018); Thurston et al. (2016); Yang et al. (2023); Wang et al. (2015); Liu et al. (2017); Tian et al. (2018); Huang et al. (2019); Wang et al. (2019); Wang et al. (2018); Bloom et al. (2015); Huang et al. (2014); Deng et al. (2022); Han et al. (2014) [13, 35, 47, 61–63, 65, 70, 71, 73, 75, 78–80, 82, 83, 87–90, 93, 95]	Concurrent: Single spot urine sample collected on the same day as semen analysis. Reflects recent exposure (hours/days) rather than the full 70–90-day spermatogenic window.
Serum (Blood)	Cross-Sectional	Specht et al. (2014); Wang et al. (2019); Axelsson et al. (2015) [66, 81, 88]	Concurrent: Single blood draw concurrent with semen sampling. Serum levels reflect steady-state burden but may vary from urinary excretion profiles.
Semen (Seminal Plasma)	Cross-Sectional	Yuan et al. (2021); Wang et al. (2016); Pant et al. (2014) [77, 84, 86]	Direct Tissue: Measurement of metabolites directly within the reproductive compartment (seminal plasma) concurrent with sperm analysis.
Mixed / Indirect	Registry / Modeling	Broe et al. (2018) [68]	Pharmacological: Exposure inferred from prescription registry data for phthalate-coated medications (high-dose exposure).
		Ramsay et al. (2023) [72]	Environmental: Exposure inferred from industrial air pollution modeling rather than direct biospecimen quantification.

### Methodological heterogeneity in semen analysis and reference standards

The included studies exhibited considerable variation in the laboratory methods used for semen evaluation and the reference standards applied to define normality as shown in Table 3.

Of the 38 included studies, manual microscopic examination was used for determining sperm concentration and motility (*n* = 16), while Computer-Assisted Semen Analysis (CASA) was utilized in (*n* = 21) studies to enhance objectivity and provide kinematic data. Notably, the reference limits used to categorize semen quality shifted significantly over the study period, reflecting the evolution of World Health Organization (WHO) guidelines. Early studies primarily adhered to the WHO 5th (2012) and 6th (2021) editions, while some also cited the 4th edition (1999) [95, 96]. The most pronounced shift was observed in sperm morphology assessment, where studies shifted from various “amorphous” criteria to the “Kruger Strict Criteria” mandated by later WHO editions.

Regarding mediating endpoints, specifically Sperm DNA Fragmentation (SDF), we observed a lack of harmonized definitions and methodologies. Studies reported DNA integrity using diverse assays, including the Sperm Chromatin Structure Assay (SCSA), TUNEL, and Sperm Chromatin Dispersion (SCD). These assays utilize different biological principles ranging from acid-induced denaturation to direct strand-break labeling resulting in non-interchangeable Sperm DNA Fragmentation Index (DFI) values across the literature. This methodological diversity across WHO versions, analytical platforms (CASA vs. Manual), and SDF assays represents a primary source of the observed clinical heterogeneity in this review.

**Table 3:** Summary of Analytical Methods and WHO Manual Versions Used Across Included Studies

Study (Author, Year)	Semen Analysis Method (CASA / Manual)	WHO Manual Edition (Year)	SDF Assay Method (if performed)
Al-Saleh et al., 2019 [74]	Manual (Microscopic)	5th Edition (2010)	Comet Assay
Al-Saleh et al., 2019 [95]	Manual (Microscopic)	5th Edition (2010)	N/A
Albert et al., 2018 [75]	CASA (Hamilton Thorne)	5th Edition (2010)	N/A
Axelsson et al., 2015 [81]	Manual (Microscopic)	4th Edition (1999)	N/A
Bloom et al., 2015 [35]	CASA (IVOS)	4th Edition (1999)	N/A
Broe et al., 2018 [68]	Manual (Microscopic)	5th Edition (2010)	N/A
Caporossi et al., 2023 [63]	Manual (Microscopic)	5th Edition (2010)	N/A
Chang et al., 2017 [94]	CASA (Hamilton Thorne)	4th Edition (1999)	N/A
Chen et al., 2017 [76]	Manual (Microscopic)	5th Edition (2010)	N/A
Den Hond et al., 2015 [70]	Manual (Microscopic)	4th Edition (1999)	N/A
Deng et al., 2022 [90]	CASA	5th Edition (2010)	N/A
Gao et al., 2024 [61]	CASA (SCA Scope)	6th Edition (2021)	N/A
Han et al., 2014 [93]	Manual (Microscopic)	4th Edition (1999)	Comet Assay
Huang et al., 2014 [47]	Manual (Microscopic)	4th Edition (1999)	TUNEL Assay
Huang et al., 2019 [87]	Manual (Microscopic)	5th Edition (2010)	N/A
Jurewicz et al., 2016 [73]	Manual (Microscopic)	4th Edition (1999)	N/A
Liu et al., 2017 [82]	CASA (WLJY 9000)	4th Edition (1999)	N/A
Liu et al., 2019 [62]	CASA (WLJX 9000)	5th Edition (2010)	N/A
Mínguez-Alarcón et al., 2018 [85]	CASA (Hamilton Thorne)	5th Edition (2010)	N/A
Nassan et al., 2016 [69]	CASA (Hamilton Thorne)	5th Edition (2010)	N/A
Pan et al., 2015 [65]	CASA	5th Edition (2010)	SCSA
Pan et al., 2016 [67]	CASA (CFT-9201)	5th Edition (2010)	N/A
Pant et al., 2014 [84]	Manual (implied by text)	5th Edition (2010)	Comet Assay
Ramsay et al., 2023 [72]	Manual (Ref. Lab)	Mixed (4th & 5th Editions)	N/A
Rana et al., 2020 [64]	Manual (Microscopic)	4th Edition (1999)	N/A
Specht et al., 2014 [66]	Manual (Neubauer/Microscopic)	4th Edition (1999)	N/A**
Sun et al., 2020 [98]	CASA (Hamilton Thorne)	5th Edition (2010)	N/A (Measured Ca2+, Tyrosine Phos.)
Thurston et al., 2016 [78]	Manual (MicroCell)	4th Edition (1999)	N/A
Tian et al., 2019 [83]	CASA (WLJY 9000)	5th Edition (2010)	N/A (Measured DNA Methylation)
Wang et al., 2015 [80]	CASA (WLJX 9000)	5th Edition (2010)	N/A
Wang et al., 2016 [86]	CASA (WLJX 9000)	5th Edition (2010)	N/A
Wang et al., 2016 [99]	N/A (Focus on DNA/Apoptosis)	5th Edition (2010)	Neutral Comet Assay; Annexin V (Apoptosis)
Wang et al., 2018 [89]	CASA	5th Edition (2010)	N/A
Wang et al., 2019 [88]	CASA	5th Edition (2010)	N/A
Wang et al., 2020 [60]	Standardized (not specified as CASA)	5th Edition (2010)	N/A
Yang et al., 2017 [71]	CASA	5th Edition (2010)	Annexin V (Apoptosis)
Yang et al., 2023 [79]	CASA	5th Edition (2010)	N/A (Measured ROS, MMP, Ca2+)
Yuan et al., 2021 [77]	CASA (SCA)	5th Edition (2010)	N/A (Measured Steroids)

### Quality assessment

**Fig. 2:**
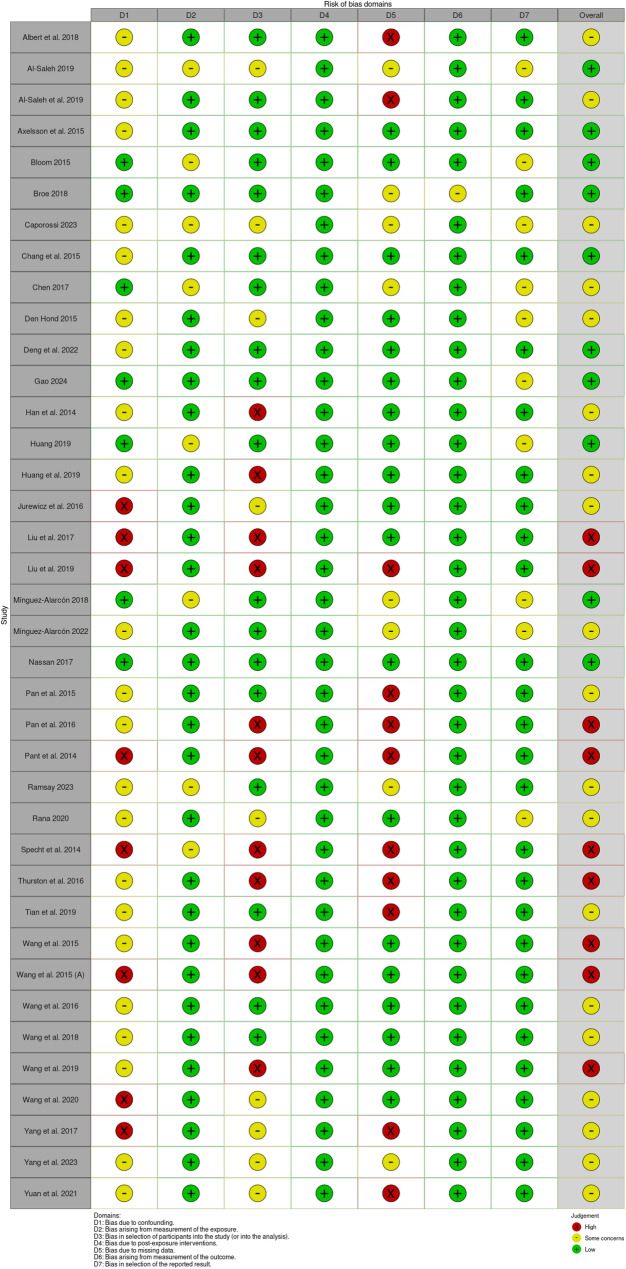
Traffic light plot for the ROBINS-E tool for risk of bias assessment for all the studies

The risk of bias was assessed via Robins-E tool [97] for 25 cross-sectional studies, 7 prospective studies, 5 case‒control studies and one retrospective cohort study. The risk of bias assessed via the ROBINS-E tool was created via the Robvis (visualization tool) [98], as shown in Fig. 2.

**Fig. 3:**
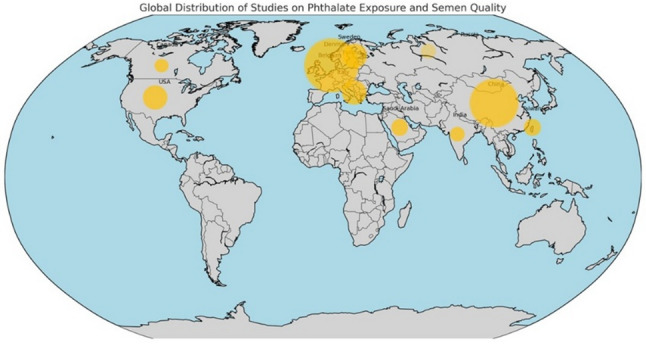
Distribution of studies from different parts of the world

A figure showing the distribution of studies conducted and analysed in this review is shown in Fig. 3.

## Discussion

This systematic review synthesizes evidence from 38 studies evaluating the relationships between phthalate exposure and male reproductive parameters. Phthalates, which are ubiquitous environmental endocrine-disrupting chemicals (EDCs), are widely used in plastics, pharmaceuticals, and personal care products. The findings across diverse populations and study designs converge on a clear signal: phthalates detrimentally affect semen quality, with varying degrees of severity influenced by exposure timing, dose, and individual susceptibility. This review specifically examines key domains, such as sperm concentration, motility, morphology, volume, hormonal disruption, oxidative stress, and epigenetic mechanisms.

**Fig. 4:**
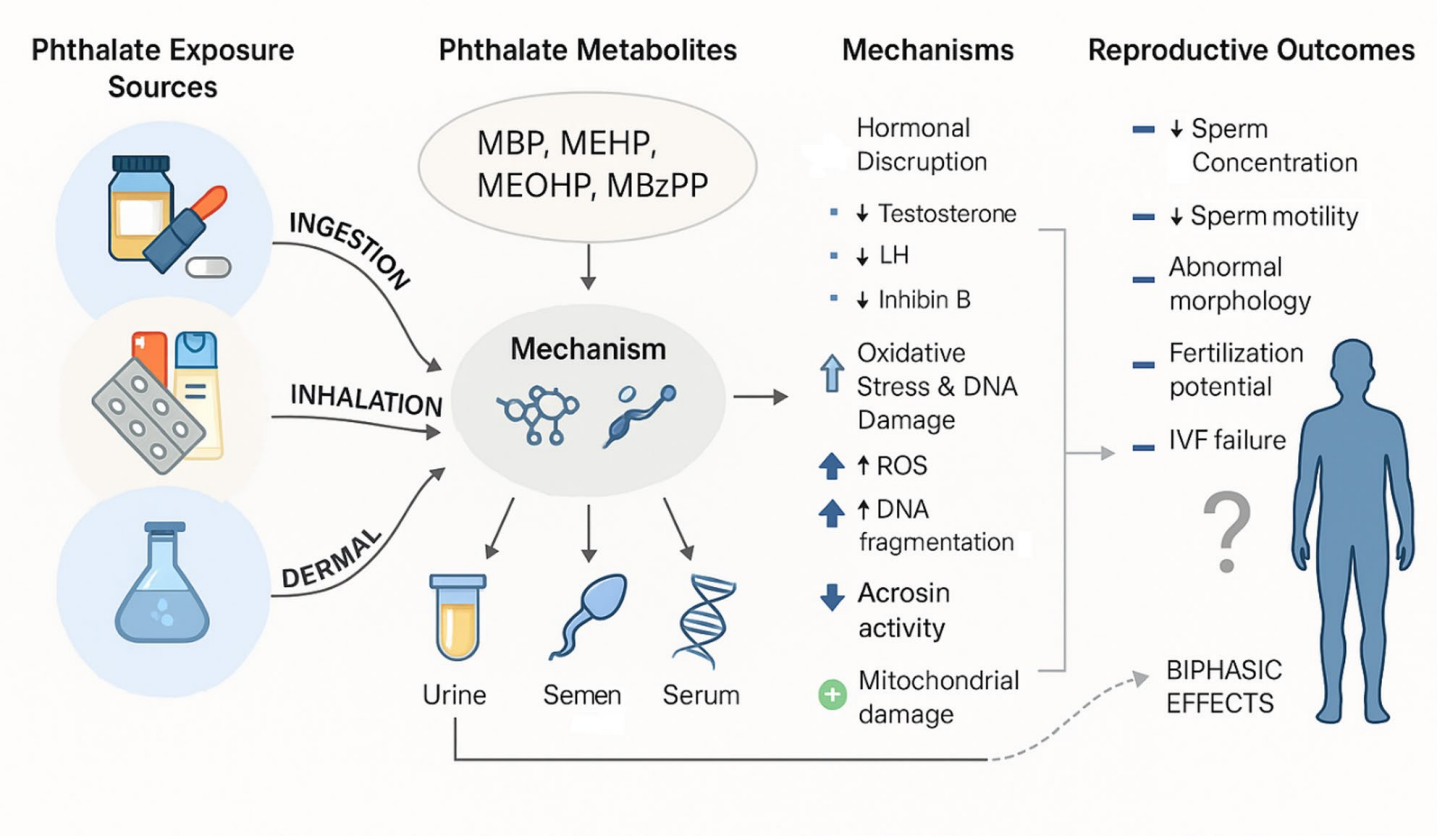
Mechanisms linking phthalate exposure to impaired male reproductive health

Figure 4 illustrates the pathways through which phthalate exposure affects male reproductive health from environmental sources and metabolites to mechanisms such as hormonal disruption, oxidative stress, and epigenetic changes. These factors culminate in reduced sperm concentration, motility, morphology, and semen volume. The diagram also highlights modifying factors such as genetics and nutrition, offering a visual summary of the complex interplay explored in the sections that follow.

### Mechanisms

Phthalates exert their deleterious effects through site-specific toxicity across the male reproductive tract, targeting the testis, epididymis, and accessory glands. In the testis, Sertoli and Leydig cells are the primary targets. Phthalate metabolites, particularly MEHP, disrupt the blood-testis barrier by interfering with vimentin filaments in Sertoli cells, leading to germ cell apoptosis and sloughing [70]. Simultaneously, they impair Leydig cell function by inhibiting steroidogenic enzymes (e.g., 17β-HSD), resulting in reduced testosterone and INSL3 levels which are essential for spermatogenesis [21, 69]. Following production in the testis, sperm maturation occurs in the epididymis. Phthalates induce oxidative stress within the epididymal microenvironment, depleting antioxidant enzymes and damaging the sperm membrane, which compromises motility and fertilization potential [38]. Finally, the volume of the ejaculate is largely determined by secretions from the prostate and seminal vesicles. Since these accessory glands are highly androgen-dependent, the anti-androgenic action of phthalates (such as DBP and DEHP) reduces glandular secretory function, thereby diminishing overall semen volume [86].

### Sperm concentration and total sperm count

The sperm concentration and total sperm count were consistently negatively affected by phthalate exposure in a majority of the studies. Metabolites such as MBP, MEP, MEHP, and DEHP are most frequently implicated [60–63, 66, 72, 76, 79, 80, 84, 99, 100]. In a large Chinese cohort, a dose‒dependent relationship was observed, with the sperm concentration decreasing progressively with increasing urinary MBP and MEP levels [62]. Mediation analysis indicated that oxidative stress markers such as 8-OHdG explained a significant portion of this effect.

Notably, studies involving occupational exposures, such as PVC workers [47] and industrial employees [72], reported significant reductions in both sperm concentration and count, which aligns with earlier toxicological findings in animal models. Among young Russian men, phthalate exposure during late puberty, specifically to DiNP, was associated with a 30–32% decline in sperm count and concentration [92], underscoring the critical role of developmental timing.

Conversely, a subset of studies revealed no significant associations, particularly among fertile men in the general population [64, 75, 78, 93]. This suggests that adverse effects may be more apparent in already subfertile individuals or those with higher cumulative exposures. Additionally, some inconsistencies may arise from variability in phthalate metabolism, differences in exposure windows, and reliance on single spot urine measurements that may not reflect long-term exposure.

Interestingly, a few studies have identified paradoxical or nonmonotonic responses. For example, in Saudi men and Chinese individuals, certain DEHP metabolites (e.g., MECPP and MEHHP) are positively associated with the sperm concentration [87, 99]. These findings suggest the possibility of biphasic effects at different dose thresholds, potentially mediated by adaptive hormonal responses or metabolic compensation.

### Sperm motility

Sperm motility, especially progressive motility, is one of the most sensitive parameters disrupted by phthalate exposure. Numerous studies have revealed significant inverse relationships between exposure to DEHP, MBzP, MCPP, and MEHP and reduced sperm motility [47, 60, 61, 63, 69, 76, 79, 81, 86, 101].

In a study evaluating 55 endocrine-disrupting chemicals, MCPP emerged as the most detrimental compound for progressive motility [61]. Importantly, men with higher seminal DPA, a type of omega-3 PUFA, showed partial protection against motility impairment, indicating the potential of dietary modification as a mitigating factor.

Pharmaceutical exposure provided further evidence. In men treated with mesalamine containing DBP, motility decreases significantly and fails to recover even after exposure cessation, indicating long-lasting effects on the seminiferous epithelium [69]. Similarly, in PVC workers exposed to high DEHP levels, reduced motility was observed in tandem with increased ROS generation and sperm apoptosis [47].

Some studies linked reduced motility to sperm structural changes such as mitochondrial dysfunction and impaired ATP production. For example, the MARHCS study demonstrated that MEHP exposure led to decreased progressive motility, potentially mediated by disruptions in spermatogenic mitochondrial DNA content [79].

A mechanistic study revealed that MEHP induced premature sperm hyperactivation and spontaneous acrosome reactions via increased intracellular calcium and tyrosine phosphorylation, processes that, if activated prematurely, can compromise motility and fertilization potential [102].

Notably, a few studies reported improved motility at low levels of MEHP or DEHP [83, 87, 90], suggesting biphasic responses. One proposed mechanism is that low phthalate exposure may transiently enhance capacitation-like changes or modulate DNA methylation (e.g., LINE-1 hypomethylation) to improve sperm function. However, the potential for long-term damage even at low exposure levels underscores the need for careful interpretation.

### Sperm morphology

Sperm morphology was another domain consistently affected by phthalate exposure. Elevated levels of MBP, MEHP, and MiBP are associated with abnormal sperm head shapes, tail defects, and decreased percentages of individuals with normal morphology [62, 65, 67, 80, 86, 88, 89]. In a Chinese infertility clinic study, abnormal sperm heads were significantly more common in men with higher MBP and MEHP levels, suggesting possible disruptions during spermiogenesis [80].

The reduced acrosin activity observed with MBP and MiBP exposure [65, 67] implies that impaired zona pellucida penetration is crucial for successful fertilization. Moreover, studies have documented associations between DEHP exposure and increased high DNA stainability (HDS), indicating the presence of sperm immaturity and chromatin defects [60, 81].

Different phthalates appear to disrupt sperm morphology through distinct pathways. DEHP and its metabolites are strongly linked to defects in the sperm head and chromatin immaturity (high DNA stainability), likely due to oxidative stress disrupting spermiogenesis [60, 81]. Conversely, MBP (a metabolite of DBP) has been specifically associated with tail defects and overall abnormal forms, potentially mediated by the disruption of cytoskeletal elements during the elongation phase of sperm development [65]. This suggests that while oxidative stress is a common mechanism, the specific structural abnormality may depend on the timing and type of phthalate exposure during the spermatogenic cycle.

Morphological alterations are also linked to thyroid hormone disruptions. Free thyroxine (FT4) and TSH were found to mediate the relationship between MEPs and abnormal morphology [89], suggesting a systemic hormonal pathway through which phthalates influence sperm development. Nevertheless, not all studies reported significant findings. Those focusing on healthy or low-exposure populations sometimes fail to detect changes in morphology, possibly due to threshold effects, better compensatory mechanisms, or differences in semen analysis protocols [64, 75, 78].

### Semen volume

Semen volume was among the most inconsistently affected parameters. Several studies reported a reduced volume with increased levels of DEHP or MBP metabolites [66, 67, 86]. For example, in a European cohort, a 5.3% decline in semen volume was observed in men in the highest quartile of Proxy-MEHP exposure [66]. Similar trends were reported in the MARHCS study, where increased MEP and MEHP levels were associated with lower ejaculate volume [76]. Mechanistically, reductions in semen volume may reflect impaired secretory function of accessory glands (prostate, seminal vesicles), possibly via androgen depletion or local tissue damage induced by phthalate metabolites.

The reduction in semen volume appears to be driven by specific phthalate congeners that exhibit strong anti-androgenic activity. Studies indicate that metabolites of DEHP (e.g., MEHP, MEHHP) and DBP (e.g., MBP) are most consistently associated with reduced ejaculate volume [66, 86]. This is likely due to their suppression of accessory gland function (seminal vesicles and prostate), which contributes the majority of seminal fluid. In contrast, low molecular weight phthalates like MEP, often found in personal care products, show inconsistent associations with volume, suggesting that the “anti-androgenic potency” of the specific phthalate type is a critical determinant of this outcome.

However, several studies reported no effect on volume despite marked impairments in other parameters, such as motility and morphology [61, 64, 75, 78]. This may indicate that semen volume is less sensitive to phthalates or that the exposure window required to affect volume may differ from that affecting sperm production. Additionally, the role of hydration status and abstinence could confound volume-related findings.

### Hormonal disruption and endocrine mechanisms

A central mechanism underlying the reproductive toxicity of phthalates is endocrine disruption. Multiple studies have reported significant reductions in serum testosterone, luteinizing hormone (LH), and inhibin B following phthalate exposure [65, 66, 69, 70, 76, 99, 100]. In a crossover–crossback study of IBD patients, men newly exposed to DBP-containing mesalamine experienced a 13.9% decrease in LH and marginal reductions in testosterone and FSH levels, suggesting suppression of the hypothalamic‒pituitary‒gonadal (HPG) axis [69]. The reversal of these changes upon cessation of exposure further reinforces the causal role of DBP.

Inhibin B suppression, a marker of Sertoli cell function, is consistently associated with MEHP and MBP [65, 70]. This is critical because Sertoli cells support developing germ cells and are essential for maintaining spermatogenesis. Disruptions in androgen precursors have also been documented. For example, MnBP was inversely associated with sperm motility via reduced androstenedione levels [77]. Thyroid hormone alterations have been shown to mediate changes in both morphology and motility, expanding the hormonal axis involved in phthalate-induced reproductive dysfunction [89].

In rare cases, low-dose exposure is associated with mild increases in androgen activity or sperm function, potentially due to transient compensatory mechanisms, although the long-term effects remain unclear [82, 83].

### Oxidative stress, DNA damage, and apoptosis

Oxidative stress and genotoxicity are increasingly recognized as key mediators of phthalate-induced reproductive harm. Many studies have reported elevated levels of reactive oxygen species (ROS), lipid peroxidation products (e.g., malondialdehyde), and DNA fragmentation (measured as DFI or comet assay tail length) in association with phthalate metabolites [47, 62, 65, 79, 88, 99, 101]. Elevated DNA fragmentation is associated with reduced motility, abnormal morphology, and lower fertilization success in IVF settings [99]. For example, increased ROS generation and sperm apoptosis are significantly greater in men exposed to high levels of MEHP and MEHHP [47].

Phthalate-induced oxidative stress also affects sperm mitochondria, as evidenced by altered mtDNA copy number, which results in a reduction in the sperm concentration [79]. In parallel, inflammatory pathways and altered antioxidant enzyme activity (e.g., reduced catalase) have been documented [99]. Furthermore, polymorphisms in apoptosis-regulating genes such as Fas, FasL, and caspase3 amplified the detrimental effects of phthalates, suggesting that gene‒environment interactions modulate risk [71, 72].

### Non-monotonic dose-response and biphasic effects

While the prevailing toxicological model assumes a linear relationship where higher exposure equates to greater toxicity, our review identified distinct non-monotonic dose-response (NMDR) patterns in several studies, a hallmark of endocrine-disrupting chemicals. For instance, Deng et al. [90] observed an inverted U-shaped relationship where moderate exposure to DEHP was associated with increased sperm progressive motility, whereas higher concentrations led to significant declines. Similarly, Tian et al. [83] and Huang et al. [47] reported paradoxical positive associations between low-level phthalate exposure and sperm motility or concentration.

These biphasic responses likely reflect hormesis, an adaptive biological response where low-dose stress stimulates compensatory mechanisms such as increased mitochondrial activity or antioxidant upregulation before toxic thresholds are breached. Specifically, Tian et al. [83] proposed that low-level exposure might induce specific DNA methylation changes (e.g., LINE-1 hypomethylation) that transiently enhance sperm function. However, Al-Saleh et al. [74] noted that while certain DEHP metabolites (e.g., MECPP) appeared protective for sperm concentration in specific subsets, this might also reflect receptor saturation dynamics or differential activation of estrogenic versus androgenic pathways at varying doses.

These findings have critical implications for regulatory policy. Traditional risk assessments often rely on “no observed adverse effect levels” (NOAEL) derived from high-dose testing, assuming safety below a linear threshold. The evidence of NMDR suggests that “safe” levels established by linear extrapolation may overlook adverse or disruptive effects occurring at environmentally relevant low doses. Consequently, future policy frameworks must incorporate low-dose testing protocols to accurately capture the complex risk profiles of phthalates.

### Impact of analytical methods and standards on study heterogeneity

A significant challenge in synthesizing the results of phthalate exposure studies on semen quality is the profound heterogeneity introduced by differences in analytical methodologies and the evolution of international standards over the study period.

### Heterogeneity due to WHO manual editions

The World Health Organization (WHO) has periodically updated its *Laboratory Manual for the Examination and Processing of Human Semen*, with the 5th (2012) [95] and 6th (2021) [96] editions being most relevant to the included studies, however some studies also cited the 4th edition in their studies.

A major source of non-comparability lies in the shifting reference values for key semen parameters:

- *Morphology*: The 5th edition drastically changed the normal cut-off for sperm morphology to 4% (strict criteria), a substantial reduction from the > 14% criteria often used under the 4th edition. Studies published before 2010 that used previous editions or non-strict criteria may report a higher prevalence of “normal” samples compared to later studies, directly influencing the association between phthalate exposure and morphology-related outcomes.
- *Motility*: The classification and cut-offs for motility have also been adjusted across editions, with the progressive motility cut-off changing from 32% (5th Ed.) to 30% (6th Ed.), which can subtly impact the classification of oligo/asthenozoospermia.

The varied use of these editions often published years apart from the study’s data collection means that the definition of an “abnormal” semen parameter is inconsistent across the systematic review, contributing substantially to statistical and clinical heterogeneity.

### Heterogeneity due to semen analysis method (CASA vs. Manual)

The method employed for semen analysis is another critical source of heterogeneity. Manual microscopic assessment, while cost-effective and feasible, is inherently subjective and prone to significant inter-observer variability [103].

In contrast, Computer-Assisted Semen Analysis (CASA) systems offer greater objectivity, standardization, and the ability to capture detailed sperm kinematics (velocity, linearity) which are unavailable by manual methods. However, CASA is not without its limitations as different CASA software systems utilize varied algorithms and calibration settings, leading to differences in measured outcomes, even for the same sample. Secondly, CASA systems may sometimes overestimate sperm concentration due to the misidentification of non-sperm debris, or the results may be unreliable in very low- or high-concentration samples [104].

Studies that rely on manual methods are susceptible to technician-related bias, while studies using CASA systems must be compared cautiously due to potential variations between the specific hardware and software used. We have summarized the analytical method used by each study in Table 3 to allow readers to better assess this analytical heterogeneity.

### Lack of harmonized definitions for Sperm DNA Fragmentation (SDF)

Sperm DNA fragmentation (SDF), often measured as the DNA Fragmentation Index (DFI), is an emerging mediating endpoint in male fertility that is increasingly evaluated in phthalate-exposure studies [105]. However, the inclusion of SDF testing introduces additional analytical noise due to a lack of assay harmonization.

Several common assays are used to measure SDF, including the Sperm Chromatin Structure Assay (SCSA), the TUNEL (Terminal deoxynucleotidyl transferase dUTP Nick-End Labeling) assay, and the Sperm Chromatin Dispersion (SCD) test [106, 107]. The issue is that each assay measures a slightly different aspect of DNA integrity (e.g., SCSA measures susceptibility to acid denaturation, while TUNEL and SCD directly detect breaks) [106]. Also unstandardized cut-offs that is aside from SCSA, which has clinically validated thresholds, most SDF tests lack standardized cut-off values for defining “high” versus “low” fragmentation, with each laboratory or study setting its own standards. Consequently, direct comparison of DFI values or the prevalence of ‘high SDF’ across studies using different methodologies (e.g., comparing a study using TUNEL to one using SCSA) should be undertaken with extreme caution, as the numerical results are not interchangeable.

### Limitations of the included evidence

The strength of the evidence is moderated by several limitations. First, the predominance of cross-sectional studies limits causal inference. Most studies measured both exposure and outcome at a single time point, which complicates assessments of temporality. While prospective cohort studies offer a more rigorous approach, they remain rare and are often limited by sample size.

Second, exposure misclassification is a concern. Phthalates have short biological half-lives, and their urinary metabolites reflect only recent exposure. Single spot urine or serum samples may not adequately represent chronic exposure, particularly in the context of spermatogenesis, which spans approximately 70–90 days. Although creatinine or specific gravity adjustments are commonly used, within-subject variability remains an issue.

Third, semen analysis and hormone assays were not standardized across studies. While some studies adhered to WHO guidelines and used computer-assisted semen analysis (CASA), others relied on manual counting or clinical ICD codes. Outcome heterogeneity complicates comparisons and limits opportunities for meta-analysis. Moreover, not all studies have measured advanced endpoints, such as sperm DNA fragmentation, oxidative stress markers, or epigenetic changes. Fourth, confounding factors are variably addressed. While most studies adjusted for common confounders such as age, BMI, smoking, and abstinence time, fewer accounted for occupational exposures, dietary patterns, socioeconomic status, or coexposure to other endocrine-disrupting chemicals (EDCs) such as bisphenols, organophosphate esters, and microplastics. Some studies have explored gene environment interactions, but these studies are underpowered and inconsistent. Selection bias is also a concern, particularly in clinic-based studies where participants may not represent the general population. Confounding factors were variably addressed across the included studies, influencing the strength of the evidence. The majority of the reviewed studies [35, 62, 80, 99] consistently adjusted for core confounders including paternal age, body mass index (BMI), smoking status, and sexual abstinence time. Several high-quality cohorts further adjusted for covariates such as alcohol consumption, race/ethnicity, recent fever, and socioeconomic indicators (education/income) [35, 62]. However, heterogeneity remains a limitation; fewer studies accounted for environmental co-exposures (e.g., bisphenols, heavy metals), dietary patterns, or occupational hazards beyond specific plasticizer handling. Despite these variations in statistical adjustment, the negative associations between phthalate metabolites (particularly MEHP and MBP) and semen parameters persisted in multivariate models across diverse populations. This suggests that the observed effects are likely independent of these standard demographic and lifestyle confounders, although the residual influence of unmeasured environmental co-exposures cannot be fully ruled out.

Finally, a few studies have suggested nonmonotonic dose‒response relationships, particularly at low exposure levels. These findings, while biologically plausible in the context of endocrine disruption, challenge conventional toxicological assumptions and require further investigation.

### Limitations of the review processes

Our review methodology has several limitations. We included only peer-reviewed articles published in English, which introduces potential language and publication bias. Unpublished data, conference abstracts, and gray literature were excluded, potentially omitting relevant findings.

Owing to high heterogeneity in exposure metrics, outcome definitions, and statistical models, we did not conduct a formal meta-analysis. This decision was made to preserve the validity of our synthesis but limits the ability to provide pooled effect estimates. Instead, we opted for a narrative synthesis supplemented by risk of bias assessment using ROBINS-E, which provided a structured approach to evaluating study quality. Risk of bias judgments were made via a balanced interpretation of the ROBINS-E domains, but some degree of subjectivity is inherent to these assessments. For example, the classification of exposure as low or moderate risk often depends on the availability and timing of biospecimen collection, which is not uniformly reported.

### Implications for practice, policy, and future research

The findings of this review have implications for clinical practice, public health policy, and future research. Clinicians, particularly those in reproductive medicine, should be aware of environmental exposures, such as phthalates, that may impact semen quality and hormonal balance. While individual-level interventions are limited, counselling patients on avoidable sources such as phthalate-containing personal care products, food packaging, and certain medications may be advisable, especially for men with unexplained infertility.

From a policy perspective, these findings add to the growing body of evidence supporting stricter regulation of phthalates in consumer goods and pharmaceuticals. Several high-molecular-weight phthalates remain in widespread use despite mounting evidence of reproductive toxicity. Regulatory agencies should consider cumulative risk frameworks that account for simultaneous exposure to multiple EDCs. Workplace standards in industries involving PVC, solvents, or chemical manufacturing may also require updating to protect workers at elevated risk.

Research priorities should include large-scale, longitudinal studies that capture exposure and outcome data across key reproductive windows. Incorporating repeated measures of phthalate metabolites and semen parameters would improve exposure assessment and temporal resolution. Studies should also evaluate the impact of interventions such as dietary modification, supplementation with antioxidants, or the use of phthalate-free products on reproductive endpoints. Additionally, mechanistic studies linking human biomonitoring to oxidative stress, epigenetic changes, and sperm transcriptomics are needed to strengthen causal inference. The role of genetic susceptibility in modulating phthalate effects remains underexplored and warrants further investigation. Advanced statistical methods, including mixture modelling and mediation analysis, are critical for dissecting complex exposure‒outcome relationships. To address the reality of multi-pollutant exposure, future research should move beyond single-chemical models and employ mixture modeling approaches, such as Weighted Quantile Sum (WQS) regression or Bayesian Kernel Machine Regression (BKMR). These methodologies allow for the evaluation of the joint effects of chemical mixtures, helping to identify the specific contribution of individual phthalates to reproductive toxicity while accounting for the correlation structure between co-occurring EDCs.

Ultimately, the pervasive nature of phthalate exposure and its potential to impair male fertility underscore the importance of continued surveillance, regulation, and research in this area. Public health messaging, clinical awareness, and multidisciplinary collaboration are essential to mitigate the reproductive risks posed by these ubiquitous environmental contaminants.

## Conclusion

This systematic review synthesizes evidence from 38 human studies examining the impact of phthalate exposure on male reproductive health. These findings consistently demonstrate that exposure to phthalate metabolites, especially MEHP, MBP, MEP, and DEHP, is associated with significant impairments in semen quality, including reduced sperm concentration, motility, and morphology, along with altered semen volume and hormonal profiles. Mechanistic pathways such as endocrine disruption, oxidative stress, DNA fragmentation, and epigenetic alterations appear central to these effects. Based on the prevalence of cross-sectional and observational data, the overall certainty of the evidence is graded as low to moderate grade. While dose-response relationships observed in several studies strengthen the plausibility of a biological link, the lack of randomized controlled trials limit definitive causal inference.

Importantly, the review highlights potential modifiers of vulnerability, including genetic factors, age at exposure, and coexisting nutritional or environmental influences. From a precautionary standpoint, these findings underscore the need for stricter regulatory policies, improved public awareness, and targeted interventions to reduce phthalate exposure in vulnerable populations. Further longitudinal and mechanistic studies are essential to clarify dose‒response relationships, long-term fertility outcomes, and the reversibility of these effects. Protecting male reproductive health requires the integration of environmental monitoring with clinical evaluation and public health action.

## Supplementary Information

Supplementary Material 1.

## Data Availability

Data sharing is not applicable to this article as no datasets were generated or analysed during the current study.

## Abbreviations

ART: Assisted Reproductive Technology
BMI: Body Mass Index
CASA: Computer-Assisted Sperm Analysis
DBP: Dibutyl Phthalate
DEHP: Di(2-ethylhexyl) Phthalate
DEHT: Di(2-ethylhexyl) Terephthalate
DFI: DNA Fragmentation Index
DINCH: Diisononyl Cyclohexane-1,2-dicarboxylate
DNA: Deoxyribonucleic Acid
EPHPP: Effective Public Health Practice Project
FDA: Food and Drug Administration
FSH: Follicle-Stimulating Hormone
HPG: Hypothalamic–Pituitary–Gonadal axis
HPLC: High-Performance Liquid Chromatography
HR: Hazard Ratio
ICSI: Intracytoplasmic Sperm Injection
IVF: In Vitro Fertilization
LH: Luteinizing Hormone
MBP: Mono-n-butyl Phthalate
MCPP: Mono-3-carboxypropyl Phthalate
MECPP: Mono(2-ethyl-5-carboxypentyl) Phthalate
MEHHP: Mono(2-ethyl-5-hydroxyhexyl) Phthalate
MEHP: Mono(2-ethylhexyl) Phthalate
MEP: Monoethyl Phthalate
NHANES: National Health and Nutrition Examination Survey
OR: Odds Ratio
PICO: Population, Intervention, Comparison, Outcome
PRISMA: Preferred Reporting Items for Systematic Reviews and Meta-Analyses
PUFA: Polyunsaturated Fatty Acids
PVC: Polyvinyl Chloride
ROBINS: Risk Of Bias In Non-randomized Studies
ROS: Reactive Oxygen Species
SDF: Sperm DNA Fragmentation
TSH: Thyroid-Stimulating Hormone
WHO: World Health Organization
